# Sole Caregiving from Afar: Unpacking Transnational Caregiving Among the Chinese One-Children in the U.S.

**DOI:** 10.1093/geroni/igaf122.1083

**Published:** 2025-12-31

**Authors:** Qi Chen, Leyi Zhou, Julian Chun-Chung Chow

**Affiliations:** Hunter College - City University of New York, Hunter College - City University of New York, New York, United States; University of California, Berkeley, Berkeley, California, United States; University of California, Berkeley, Berkeley, California, United States

## Abstract

Despite growing research on transnational caregiving, little is known about how the Chinese only-child generation—who lack sibling support and face heightened filial expectations—conceptualizes and navigates their caregiving roles from abroad. This qualitative study examines how Chinese only-children in the United States define transnational caregiving and their responsibilities as transnational caregivers. We recruited participants through social media and conducted semi-structured online interviews with 25 Chinese only-children in the U.S. who had experience providing transnational care or were currently planning care for their aging parents in China. Most of the participants were female (68%), had a graduate degree (92%), an average age of 36.5 and an average stay of 11.2 years in the US. Using interpretive phenomenological analysis approach, we identified seven dimensions of transnational caregiving: (1) bridging healthcare information gaps between the two countries, (2) purchasing and mailing medical supplies, (3) providing emotional support through daily greetings and frequent video calls, (4) prioritizing self-care to reduce parents’ worries, (5) educating and promoting healthy behaviors to prevent illnesses requiring intensive care, (6) offering financial support, and (7) coordinating long-distance care arrangement and provider communication. These findings advance theoretical understandings of caregiving in global contexts by illustrating how transnational caregiving extends beyond traditional frameworks of caregiving through the case of Chinese only-children in the U.S. The study sheds light on how geographical distance reshapes caregiving roles, emphasizing the need for a broader conceptualization of caregiving that incorporates digital communication, cross-border healthcare gaps and resource management.

